# Inhibition of Oral Fibrinogenesis Through Transforming Growth Factor-Beta/SMAD2/3 Signalling Inhibition Using Mangosteen Pericarp Extract

**DOI:** 10.7759/cureus.47899

**Published:** 2023-10-29

**Authors:** Jency Evanjelin P, Umamaheswari TN, Elumalai Perumal

**Affiliations:** 1 Department of Oral Medicine and Radiology, Saveetha Dental College and Hospitals, Saveetha Institute of Medical and Technical Sciences, Saveetha University, Chennai, IND; 2 Centre for Global Health Research, Saveetha Medical College, Saveetha Institute of Medical and Technical Sciences, Saveetha University, Chennai, IND

**Keywords:** primary cell culture, targeted drug therapy, mangosteen, fibrinolysis, herbal extract, gene expression profile, transforming growth factor-beta, oral fibrinogenesis, anti-fibrotic effect, oral submucous fibrosis (osmf/osf)

## Abstract

Background

Chewing areca nuts can result in an oral disorder known as oral submucous fibrosis (OSF), which has the potential to be cancerous. Although it is only beginning to spread to European and the North American continents, it is highly prevalent in Southeast Asia. The probability of malignant transformation from OSF is raised by chewing tobacco use. In the current research, our objective was to assess the potential anti-fibrosis effects and the ability to prevent malignant transformation through the application of mangosteen pericarp extract.

Methodology

The Ethical Approval-IHEC/SDC/OMED-2101/23/085 from the institution was obtained to conduct this ex vivo study. The cytotoxicity effect of mangosteen pericarp extract on both normal and fibrotic buccal mucosal fibroblasts originating from OSF tissues was tested. Cell proliferation and cell migration by scratch wound healing assay was examined. Dual staining was done to determine the mode of cell death. Additionally, real-time PCR was utilized to measure the expression of TGF-β/Smad2/3 signalling, α-SMA, and type I collagen gene expression.

Results

Mangosteen extract exerted higher cytotoxicity of fibrotic buccal mucosal fibroblasts compared to normal cells. Furthermore, mangosteen-receiving cells exhibited downregulation in the expression of the TGF-β/Smad2 pathway, as well as reduced expression of α-SMA and type I collagen.

Conclusion

Findings from this study suggest that mangosteen could serve as a promising agent for averting the progression of oral fibrogenesis and halting the malignancy of the oral epithelium in patients with OSF.

## Introduction

Chewing areca nuts (arecoline) may cause oral submucous fibrosis (OSF), a condition with the potential to evolve into cancer. While it's beginning to spread to Europe and North America, it's already highly prevalent in Southeast Asia [1,2].

Fibrosis and the hardening of tissue beneath the surface are the primary noticeable features of OSF from a clinical and pathological standpoint. These changes strongly affect patients' quality of life. The formation of myofibroblasts and the continuous production of smooth muscle actin (SMA) are believed to indicate the progression of fibrosis. This shift in OSF condition plays a role in changing the tissue environment and supporting the development of cancer [3]. The majority of diseases that impact the health of the oral mucosa are acquired as a result of environmental and lifestyle factors. It's equitably usual to use smokeless tobacco in a number of ways throughout India and Southeast Asia. Betel quid - a mixture of areca nut, betel leaf, tobacco, and slaked lime - is typically chewed as part of this habit. Submucous fibrosis, a distinctive clinical marker of widespread fibrosis of the oral tissues, has developed in a significant number of users as a result. Submucous fibrosis is a well-known oral potentially malignant disorder that is characterised by fibrosis of the oral mucosa (including submucosa), and people with this condition have a higher chance of developing oral cancer. It is important to highlight that, despite OSF patients quitting the habit of chewing areca nuts, the symptoms of the disease persist [4]. It is undeniable that the development of OSF disease is inheritable. There are numerous chromosomal, genetic, and molecular alterations that contribute to the etiology of OSF [5]. During the research, a higher occurrence of HLA-A10, HLA-B7, and HLA-DR3 dominance was noted among individuals with OSF [6]. Furthermore, OSF is associated with gene polymorphisms related to COL1A1, COL1A2, COLase, LY oxidase, TGF-1, and cystatin C [7,8]. The pathogenesis of OSF has been elucidated by the roles of growth factors and cytokines released by inflammatory cells during the disease phase, which promotes fibrosis by stimulating fibroblast proliferation, increasing collagen synthesis, and decreasing collagenase production [9]. Transforming growth factor (TGF), a crucial matrix modulator, is one of these essential molecules. TGF has been identified to be associated with the regulation of cell proliferation, migration, adhesion, and apoptosis in oral submucous fibrosis [10]. Novel herbal medicines with anti-fibrotic actions are required in order to act as a targeted pharmacological therapy against specific cytokines since pro-inflammatory cytokines have a role in the OSF.

Mangosteen, the scientific name Garcinia mangostana, is known as the "queen of fruits" because it possesses characteristics that could be beneficial to the overall wellness of an individual. Since ancient times, this Southeast Asian fruit has played a crucial role in both Chinese medicine and Ayurveda. The pericarp of the mangosteen fruit is extensively used in Ayurvedic medicine to treat inflammation, diarrhoea, cholera, and dysentery [11]. Numerous studies have suggested that Xanthones from the mangosteen pericarp may have anti-inflammatory, antioxidant, and anti-apoptotic activities [12]. The antioxidant property of the extract derived from the pericarp of mangosteen is ascribed to its capability to neutralize free radicals and safeguard cells against oxidative harm. Oxidative stress has been associated with a range of clinical illnesses, such as inflammatory disorders and cancer. Hence, the extract's antioxidative capabilities have generated considerable interest in investigating its potential therapeutic uses for mitigating disorders associated with oxidative stress. Considering the persistent difficulties in properly managing fibrosis, there has been an increased focus on the exploration of new and safe anti-fibrotic medicines. This study aims to investigate how the extract from mangosteen pericarp influences the progression of oral fibrogenesis. The primary objective of this investigation is to elucidate the possible suppressive impacts of the extract on the development of oral fibrogenesis. Additionally, the study aims to investigate the underlying molecular mechanisms, with a specific emphasis on the transforming growth factor-β (TGF-β)/Smad2/3 signaling pathway, which plays a crucial role in fibrotic processes.

Rationale

The rationale for this study is to bridge the existing knowledge gap by evaluating the inhibitory effects of mangosteen pericarp extract on oral fibrogenesis. The specific focus on the ex vivo oral fibrosis model allows for controlled experimentation, providing insights into the extract's potential therapeutic applications. In this ex vivo investigation, we aimed to assess the therapeutic efficacy of mangosteen pericarp extract as a natural cure for oral fibrogenesis using a widely recognized oral fibrosis model.

## Materials and methods

An ex-vivo study was conducted after getting Ethical Approval-IHEC/SDC/OMED-2101/23/085 from the institution.

Establishment of human primary oral submucous fibrosis (OSF) cells and normal fibroblast cells

**Figure 1:**
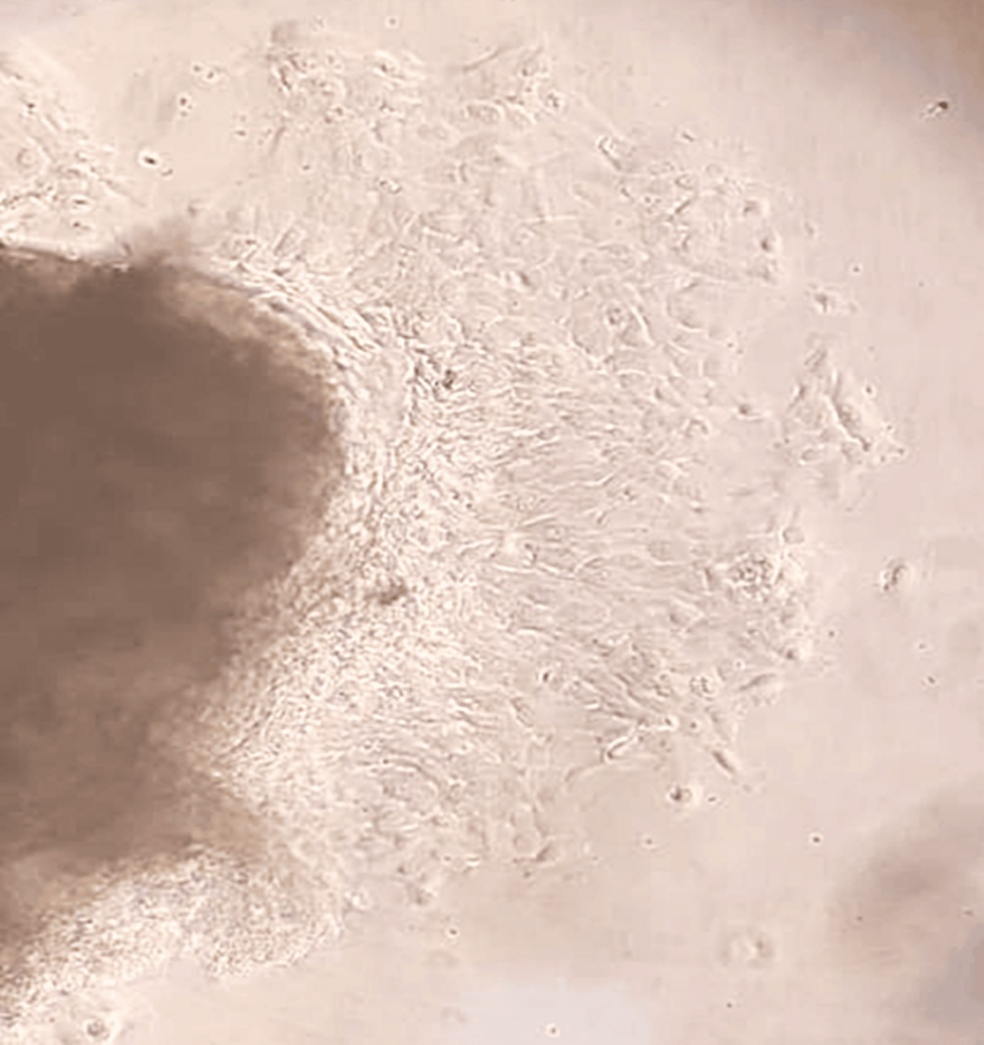
Human primary oral submucous fibrosis (OSF) cells Tissues were obtained from one clinically diagnosed OSF patient's right buccal mucosa and used for cell culture.

The Saveetha University Human Ethical Committee approved oral submucous fibrosis and gingival tissue collection procedures. Gingival tissues from healthy adolescent patients' interdental papillae were extracted during orthodontic therapy to remove their first or second premolars and used as control. For oral submucous fibrosis cells, the biopsy tissues were obtained from only one patient who was a 48-year-old male and clinically diagnosed with OSF alone included in the study. Patients who were previously under treatment for OSF were excluded from the study. Before obtaining tissue samples, patients were provided with an authorized consent form which they read and signed. The tissue was obtained from the right buccal mucosa. After the collection, the tissues were weighed (with a range of 0.18-0.21 g) and then placed in a sterile saline solution for a period of one to four hours before being prepared further. In a biosafety cabinet, tissues were sterilized before the experiments. Washing gingival tissues and oral submucous fibrosis tissues 10 times in phosphate-buffered saline (PBS) diluted their oral bacterial flora. After washing in PBS, the tissues were sliced into 1-2 mm^2^ pieces on a sterile Petri plate with culture material using a surgical blade. The collected tissues were placed into 25 cm^2^ tissue culture flasks and incubated undisturbed for 48 hours at 37°C in a humidified incubator with 5% CO_2_. After 48 hours the medium was changed. The expanded cells were used for research (Figure 1).

A density of 5×10^3^ cells per well was used to individually seed human oral submucous fibrosis and gingival fibroblast cells into 96-well plates.

The culture medium used was Dulbecco's Modified Eagle Medium (DMEM) supplemented with 1X Antibiotic Solution and 10% fetal bovine serum (Gibco, New York, USA). Next, the plates were placed within a CO_2_ incubator set at 37°C with a 5% CO_2_ environment. After being washed with 100 μL of 1X PBS, the cells were subjected to varying concentrations of mangosteen extract and then incubated at 37°C with 5% CO_2_ for a duration of 24 hours. Following this treatment, the cells’ medium was removed, and they were subsequently exposed to 0.5 mg/mL 3-(4,5-dimethylthiazol-2-yl)-2,5-diphenyltetrazolium bromide (MTT) dissolved in 1X PBS. This mixture was left to incubate for 4 hours at 37°C within the CO_2_ incubator. After the incubation period, the cells underwent a wash with 200 μL of PBS to eliminate the MTT. The crystals were then dissolved and mixed by adding 100 μL of dimethyl sulfoxide (DMSO). The absorbance was measured using microplate reader at 570 nm in which the formazan dye turned purple to blue in color.

The percentage cell viability is measured using the following formula [13]:

Cell Viability = [OD of treated cells/OD of control cells] × 100,

where OD is optical density.

Scratch wound healing assay

A total of 2×10^5^ cells of human oral submucous fibrosis were cultured in each well of six-well culture plates. A wound was created on the cell monolayer by scratching it using a 200 μl tip. The monolayer was then rinsed with PBS and subsequently imaged using an inverted microscope. The cells were subjected to treatment with mangosteen extract at a concentration of 10 μg/ml for a duration of 24 hours. The control cells, on the other hand, were exposed to a culture medium devoid of serum. Following the treatment period, the area of the wound was captured using the identical microscope. The experiments were conducted in duplicate for each treatment group.

Mode of cell death determination by acridine orange (AO)/ethidium bromide (EtBr) dual staining

Evaluation of the impact of mangosteen extract on cell death in oral submucous fibrosis was conducted using AO/EtBr dual staining. The cells were exposed to mangosteen extract (10 g/ml) for 24 hours, followed by collection and rinsing with ice-cold PBS. The pellets were redissolved in 5 mL of EtBr (1 mg/mL) and 5 mL of acridine orange (1 mg/mL). The labeled cells were then examined under a fluorescence microscope to see the apoptotic changes.

Real-time PCR

The analysis of gene expression pertaining to inflammatory markers was conducted utilizing real-time PCR. The total RNA was isolated utilizing the standardized protocol employing Trizol Reagent (Sigma-Aldrich, Germany). A total of 2 micrograms (μg) of RNA was utilized in the process of cDNA synthesis through reverse transcription, employing the PrimeScript, 1st strand cDNA synthesis kit manufactured by Takara in Japan. The genes of interest were amplified utilizing primers that were specifically designed for this purpose. The PCR reaction utilizing primer sequences was conducted using iTaq, a Universal SYBR green supermix (Bio-Rad, Hercules, CA, USA), which consists of SYBR green dye and all the necessary PCR components. Real-time PCR was conducted using a CFX96 PCR system manufactured by Bio-Rad. The results were meticulously analysed using the comparative CT method, while the fold change calculation was performed using the well-established 2-∆∆CT method, as described by the esteemed researchers Schmittgen and Livak [14].

Statistical analysis

All data obtained were analysed by One-Way ANOVA followed by Student's t-test using SPSS, represented as mean ± SD for triplicates. The level at which statistical significance was considered present was set at a p-value less than 0.05.

## Results

**Figure 2:**
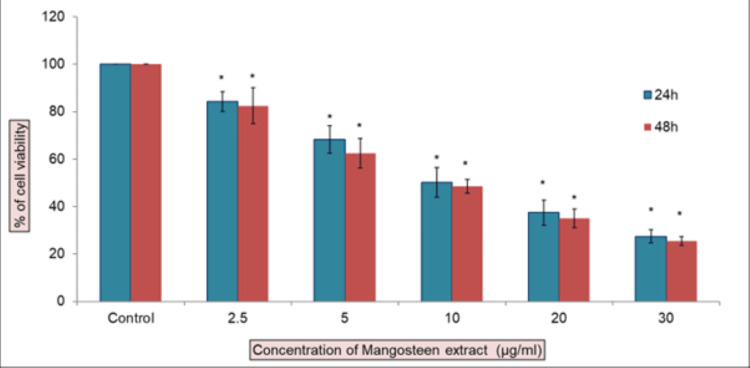
MTT assay of mangosteen extract on OSF cells at 24 hrs and 48 hrs The cytotoxic effects of mangosteen extract on oral submucous fibrosis cells. Cells were treated with mangosteen pericarp extract (2.5-30 μg/ml) for 24 h, and cell viability was evaluated by MTT assay. Data are shown as means ± SD (n = 3). * compared with the control blank group, p < 0.05.

**Figure 3:**
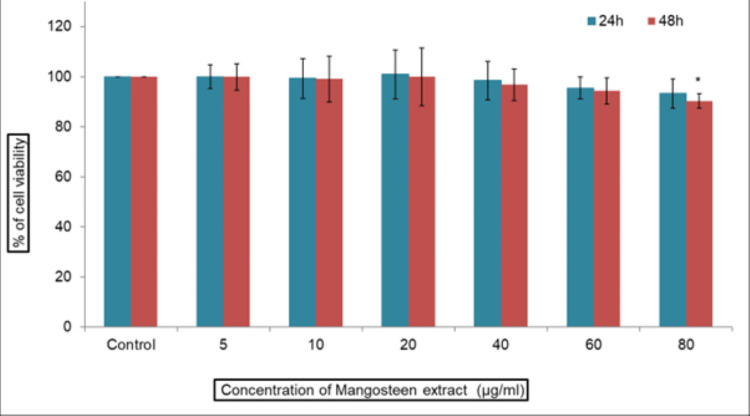
MTT assay of mangosteen extract on normal fibroblast at 24 hrs and 48 hrs The cytotoxic effects of mangosteen extract on human gingival fibroblast cells. Cells were treated with mangosteen pericarp extract (5-80 μg/ml) for 24 h, and cell viability was evaluated by MTT assay. Data are shown as means ± SD (n = 3). * compared with the control blank group, p < 0.05.

This study investigated the influence of mangosteen pericarp extract on cellular toxicity in both normal fibroblast cells and patient-derived oral submucous fibrosis cells. The cells were exposed to various extract dosages for 24 and 48 hours, and the extent of toxicity was evaluated using the MTT assay (Figures 2, 3). Our results suggest that the IC50 (Inhibitory Concentration 50) dose was attained at 10 μg/ml of extract concentration. In the MTT assay, the cell viability percentage declines as the concentration increases. We chose 10 μg/ml of concentration for further analysis.

**Figure 4:**
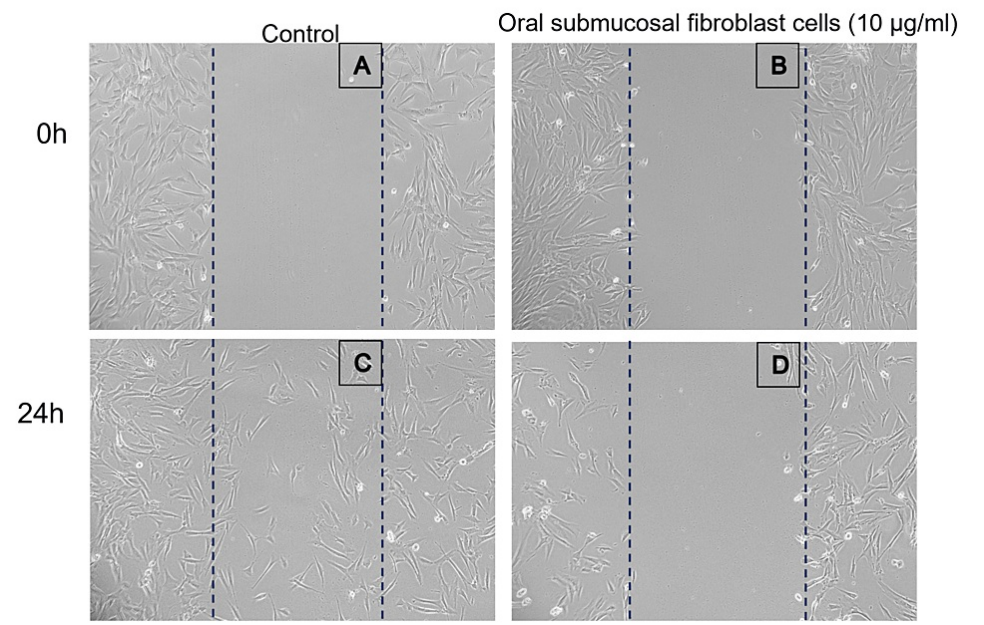
Scratch wound healing assay with 10 μg/ml concentration of mangosteen pericarp extract In vitro scratch wound healing assay. Human oral submucous fibrosis cells were injured and cell migration assay with and without mangosteen pericarp extract (10 µg/ml) treatment was performed at 24 h. Images were obtained using an inverted phase contrast microscope. The A & B panel in scratch wound healing assay shows the area of scratch created and treated with 10 μg/ml mangosteen pericarp extract, the C panel indicates the area of cell migration to the treated area, whereas the D panel indicates the cells are not migrated and there is inhibition of cell proliferation in the region which is treated with mangosteen pericarp extract.

In the scratch wound healing assay, the control, as well as oral submucous fibrosis cells, were treated with mangosteen pericarp extract where the scratch was made in the cells. The images were captured using an inverted phase-contrast microscope (Figure 4). After 24 hours of observation, there was cell migration noted in the normal cells whereas in OSF cells there was no evidence of cell migration to the treated area.

**Figure 5:**
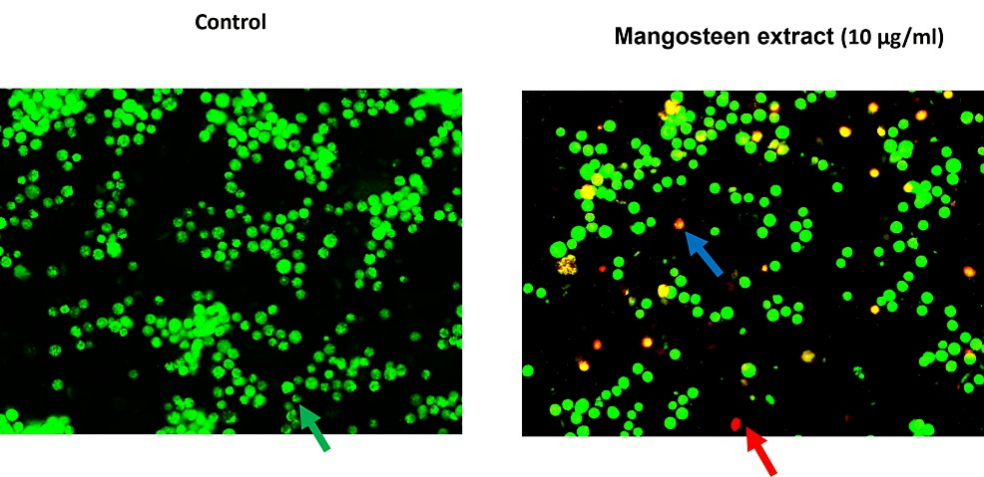
AO/EB (Acridine Orange/Ethidium Bromide) dual staining with 10 μg/ml concentration of mangosteen pericarp extract The green arrow in the dual staining figure indicates viable cells, whereas the blue and red arrows indicate early apoptotic and late apoptotic cells after being treated with 10 μg/ml concentration of mangosteen pericarp extract for 24 hours.

The cell death was determined using dual stains, where cells were treated with mangosteen extract (10 µg/ml) for 24 hours, along with a control group. Images were acquired using an inverted fluorescence microscope. The green colour indicated normal cells, yellow or orange indicated early apoptosis of cells and red indicated late apoptotic cells (Figure 5).

**Figure 6:**
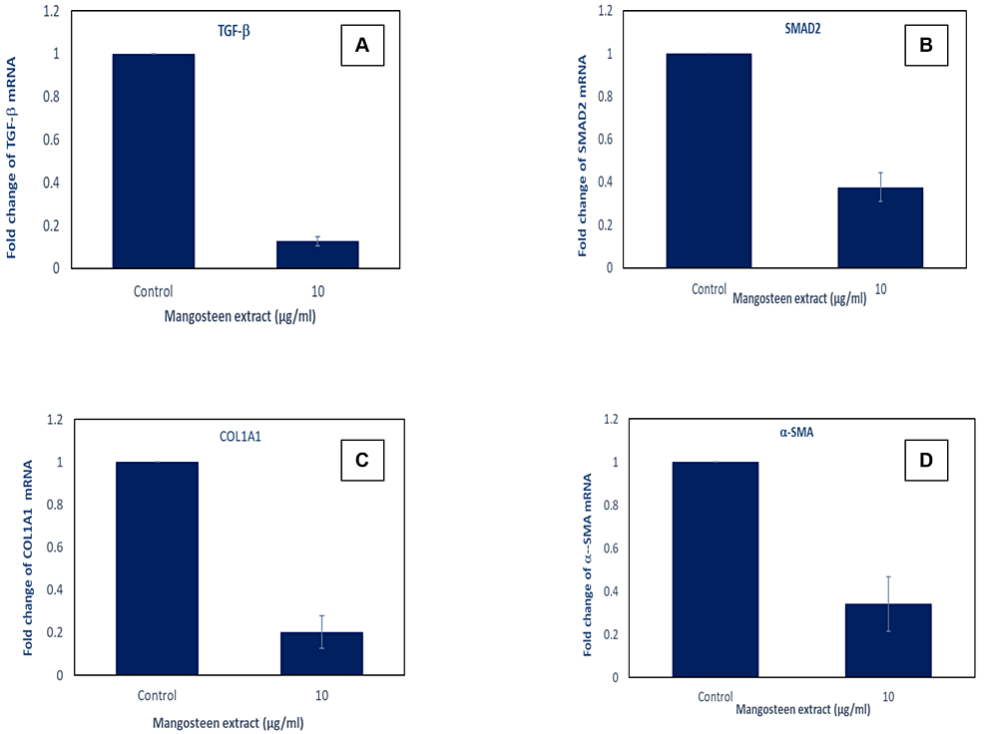
Expression of genes in mangosteen pericarp extract-treated cell lines and control Target gene expression is normalized to GAPDH mRNA expression and the results are expressed as fold change from control. Expression of A: TGF-β; B: SMAD2; C: COL1A1; D: α-SMA seen.

Furthermore, gene expression analysis was performed for transforming growth factor beta, SMAD 2 protein, COL1A1 and α-SMA. The target gene expression is standardized with respect to GAPDH mRNA expression, and the outcomes are depicted as fold changes relative to the control. Each bar on the graph represents the mean ± SEM of three independent observations, signifying statistical significance between the control and drug treatment groups at the p < 0.05 level. All the expressions seem to be downregulated when treated with mangosteen pericarp extract (Figure 6).

## Discussion

In recent years, a multitude of natural compounds, such as salvianolic acid B, curcumin, lycopene, and honokiol, have been found to impede oral fibrogenesis [15]. Clinical practice guidelines for the treatment of OSF have also mentioned the role of numerous herbal therapies but not the targeted molecular therapy which is done in our study for future applications [16]. Recent advancements in understanding the molecular composition of head and neck squamous cell carcinoma have illuminated the possibility of integrating molecular targeted therapy with established therapeutic strategies [17]. Within the myriad of factors contributing to oral cancer's origins, the intricate interplay of diverse genetic changes has urged researchers to advance their understanding. This enhanced knowledge may lead to the creation of new diagnostic and therapeutic methods for addressing oral cancer [18]. In this study, we demonstrated that mangosteen pericarp extract has the potential to alleviate various myofibroblast activities, such as cytotoxicity, cell migration, wound healing, and downregulation of gene expressions. Studies have shown that mangosteen pericarp extract possesses antifibrotic properties through multiple mechanisms. The extract from G. mangostana effectively protects against thioacetamide (TAA)-induced liver cirrhosis by significantly reducing the expression of hepatic proliferating cell nuclear antigen (PCNA), TGF-β1, and α-SMA [19]. The administration of alpha-mangostin improved cardiac hypertrophy and fibrosis in a study by Soetikno et al. [20]. α-MG demonstrates a dual effect, both restraining the multiplication of hepatic stellate cells (HSCs) and acting as a reliable indicator of fibrogenesis through the TGF-β pathway. Consequently, α-MG merits additional exploration as a potentially advantageous focal point for addressing liver fibrosis [21].

**Figure 7:**
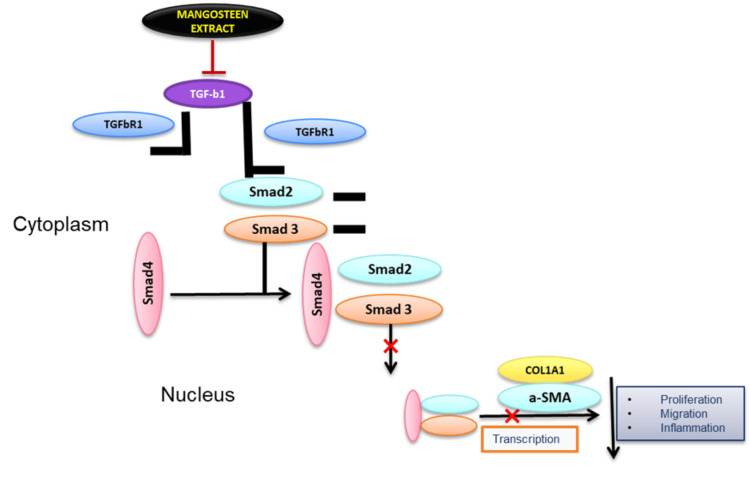
Mangosteen pericarp extract inhibiting the TGF-β/Smad2 signalling pathway Image credits: Jency Evanjelin P

The current study aimed to examine the possible anti-fibrotic properties of mangosteen pericarp extract in relation to oral fibrogenesis and its influence on the TGF-β/Smad2 signalling pathway. The results of our study indicate that the use of mangosteen pericarp extract has demonstrated efficacy in mitigating the characteristics of myofibroblasts and inhibiting the TGF-β/Smad2 signalling pathway (Figure 7).

These findings imply that mangosteen pericarp extract holds promise as a therapeutic intervention for the management of oral submucous fibrosis (OSF) and potentially as a preventive measure against the development of oral cancer.

One noteworthy finding of our study is the efficacy of mangosteen pericarp extract in attenuating myofibroblast characteristics. Myofibroblasts, which are distinguished by their ability to contract and produce extracellular matrices, are pivotal in the progression of fibrosis. Within the context of organ-specific fibrosis (OSF), the process of myofibroblast development and subsequent activation plays a substantial role in the progression of fibrosis. Our study contributes to the expanding body of evidence supporting the potential of mangosteen pericarp extract as an anti-fibrotic drug, since it demonstrates the downregulation of myofibroblast features in response to therapy.

The TGF-β/Smad2 signalling pathway is well acknowledged as a pivotal factor in the progression of fibrotic processes in different tissues, including the oral mucosa. The findings of our study present strong evidence supporting the notion that the anti-fibrotic actions of mangosteen pericarp extract are mediated, to some extent, by the modification of this particular pathway. The extract efficiently disrupts the activation of fibrogenic responses by decreasing the release of TGF-β and the nuclear translocation of Smads, thereby dampening the signalling cascade. This discovery is consistent with other research that has documented the ability of mangosteen pericarp extract to inhibit fibrosis by modulating the TGF-β signalling pathway in various tissues.

Notably, in addition to its anti-fibrotic properties, studies have indicated that the extract derived from the pericarp of the mangosteen fruit exhibits the potential in mitigating the aggressiveness of oral cancer. This observation underscores the potential of the extract not only in the management of fibrotic disorders but also in the modulation of tumorigenic processes. While our work does not delve into the specific mechanisms responsible for this anti-cancer impact, it does indicate that the extract's ability to regulate TGF-β/Smad2 signalling may have wider ramifications for the advancement of oral cancer.

The remarkable aspect lies in the potential of mangosteen pericarp extract to mitigate the pro-fibrotic effects induced by arecoline stimulation. Arecoline, a prominent alkaloid present in the areca nut, has been implicated in the aetiology of oral submucous fibrosis (OSF). The stimulation of TGF-β synthesis and subsequent initiation of fibrotic reactions have a role in the advancement of the disease. The results of our study suggest that the extract demonstrates efficacy in reducing the synthesis of TGF-β caused by arecoline, thereby accounting for its anti-fibrotic properties.

The discovery of the inhibitory effects of mangosteen pericarp extract on fibrosis, while preserving the integrity of normal fibroblasts, holds significant importance. The specificity of therapies aimed at treating fibrosis is a common difficulty, as it is crucial for anti-fibrotic medicines to exhibit selective action on diseased fibroblasts while minimising any impact on good tissue. Novel approaches are being pursued to discover potential diagnostic markers and optimize therapeutic targets, with the ultimate goal of enhancing cure rates and overall survival while reducing the financial burden on individuals [22]. The findings of our study offer initial indications that the extract derived from mangosteen pericarp exhibits potential selectivity, rendering it a desirable subject for further exploration as a potential therapeutic intervention.

Limitations

There are significant limitations associated with the utilisation of an ex vivo model for the investigation of oral fibrogenesis. Although it facilitates controlled testing and offers useful insights, it may not comprehensively depict the intricacies and dynamics of the in vivo milieu. Therefore, it is important to use caution when extrapolating the findings to real-life in vivo situations. Further work is necessary to determine the appropriate concentration that can achieve anti-fibrotic effects without causing detrimental implications on normal fibroblasts. The study presents findings that support the notion that mangosteen pericarp extract has the ability to regulate the TGF-β/Smad2 signalling pathway. However, additional research is required to comprehensively understand the specific molecular processes that contribute to its anti-fibrotic properties. Further investigations, such as gene expression profiling or proteomic analysis, may offer a more comprehensive mechanism by which the extract exerts its effects. The absence of clinical data obtained from human participants imposes constraints on the direct applicability of these findings to clinical contexts. In order to assess the safety and effectiveness of the extract in a therapeutic environment, it would be imperative to conduct clinical trials with human subjects.

Future scope

The present investigation employed an ex vivo model to examine the anti-fibrotic properties of mangosteen pericarp extract in relation to oral fibrogenesis. In order to assess the effectiveness and safety of the extract for clinical use, it is imperative to carry out in vivo investigations utilising animal models. The importance of performing well-designed clinical studies cannot be overestimated, particularly in light of the potential therapeutic benefits of mangosteen pericarp extract for oral fibrosis and its potential to impede the advancement of oral cancer. The inclusion of human patients in clinical trials would provide researchers with the opportunity to evaluate the safety, ideal dosage, and effectiveness of the extract in the treatment of oral fibrosis.

## Conclusions

In summary, our research findings indicate that the extract derived from the pericarp of the mangosteen fruit possesses anti-fibrotic properties in the context of oral fibrogenesis. This is achieved through the mitigation of myofibroblast characteristics and the inhibition of the TGF-β/Smad2 signalling pathway. Additionally, our findings suggest that it may have the potential to serve as a viable option in inhibiting the advancement of oral submucous fibrosis (OSF) towards the development of oral cancer. The results of this study justify the need for additional investigation in order to better understand the fundamental processes involved and to assess the effectiveness and safety of the extract in real-world medical environments. In conclusion, the application of mangosteen pericarp extract as an innovative therapeutic substance exhibits potential in the management of fibrotic ailments, such as OSF, and may potentially play a role in the prevention of oral cancer progression.
